# A Classification Method for the Cellular Images Based on Active Learning and Cross-Modal Transfer Learning

**DOI:** 10.3390/s21041469

**Published:** 2021-02-20

**Authors:** Caleb Vununu, Suk-Hwan Lee, Ki-Ryong Kwon

**Affiliations:** 1 Department of IT Convergence and Application Engineering, Pukyong National University, Busan 48513, Korea; exen.xmen@gmail.com; 2 Department of Computer Engineering, Dong-A University, Busan 49315, Korea; skylee@dau.ac.kr

**Keywords:** HEp-2 cell images classification, computer-aided diagnosis, deep learning, active learning, transfer learning, pattern recognition

## Abstract

In computer-aided diagnosis (CAD) systems, the automatic classification of the different types of the human epithelial type 2 (HEp-2) cells represents one of the critical steps in the diagnosis procedure of autoimmune diseases. Most of the methods prefer to tackle this task using the supervised learning paradigm. However, the necessity of having thousands of manually annotated examples constitutes a serious concern for the state-of-the-art HEp-2 cells classification methods. We present in this work a method that uses active learning in order to minimize the necessity of annotating the majority of the examples in the dataset. For this purpose, we use cross-modal transfer learning coupled with parallel deep residual networks. First, the parallel networks, which take simultaneously different wavelet coefficients as inputs, are trained in a fully supervised way by using a very small and already annotated dataset. Then, the trained networks are utilized on the targeted dataset, which is quite larger compared to the first one, using active learning techniques in order to only select the images that really need to be annotated among all the examples. The obtained results show that active learning, when mixed with an efficient transfer learning technique, can allow one to achieve a quite pleasant discrimination performance with only a few annotated examples in hands. This will help in building CAD systems by simplifying the burdensome task of labeling images while maintaining a similar performance with the state-of-the-art methods.

## 1. Introduction

The classification of the different types of the human epithelial type 2 (HEp-2) cells is one of the most important steps in the diagnosis procedure of autoimmune disease [1]. Performing this classification manually represents an arduous task and can cost a lot of time during the diagnosis process. Moreover, the manual analysis of the HEp-2 cell patterns poses a certain problem in terms of consistency of the diagnosis results, since the complexity of the images complicates the task for the pathologists [2]. This is the reason why the automatic discrimination of the different types of the HEp-2 cell images is more than necessary in order to help pathologists during the diagnosis procedure. Which makes the classification of these cells to be one of the important parts of the computer-aided diagnosis systems.

Different methods have been presented for this task in the literature. As a pattern recognition problem, the classification of the HEp-2 cells is usually tackled with a feature extraction part followed by a discrimination process. Feature extraction consists of extracting or selecting the information that is supposed to help differentiating the different cellular types. The second part of the process consists of utilizing the extracted features as the inputs of a discriminator (a classifier). Different hand-crafted features have been proposed for this purpose and many of them can be seen in the review by Foggia et al. [3]. Various descriptors like the discrete cosine transform [4,5], the scale-invariant feature transform [5,6], the local binary patterns [7,8,9] or many other different statistical features have been highlighted in the literature [10,11,12,13,14,15]. A multiclass support vector machine (SVM) is mostly used as the discriminator for these methods.

The automatic feature learning process afforded by deep learning has largely supplanted the use of these handcrafted features. In addition to the fact that the subjective choice of the features was a disadvantage for these methods in terms of consistency, their limitations in terms of the discrimination results explain why they have fallen out of use and been supplanted by the deep learning-based methods. In fact, currently, the quasi majority of the works in the literature utilize these methods in order to demonstrate their superiority over the conventional handcrafted features.

One of the pioneers works to adopt the convolutional neural network (CNN) for the HEp-2 cell classification was the method proposed by Foggia et al. [2] at the International Conference on Pattern Recognition (ICPR) HEp-2 cells classification contest in 2012. Since then, multiple works have proposed the use of CNN models in many different ways [16,17,18,19,20,21]. Among the most noticeable, Li et al. [22] have presented a customized CNN model, called the deep residual inception network (DRI-Net), which associates the residual connection from the ResNet [23] and the “Inception modules” utilized in the GoogleNet [24]. Additionally, among the most noticeable, Shen et al. [25] have used the ResNet approach but with a much deeper residual module with several cross connections between the layers. Their model was named the deep-cross residual network (DCR-Net) and they have tested a huge data augmentation process in order to boost the classification accuracy.

Interestingly, Majtner et al. [26] have proposed the use of generative adversarial networks [27], the deep convolutional generative adversarial networks (DCGAN) [28] specifically, in order to generate realistic artificial HEp-2 images. The goal was to augment the existing datasets with the artificial images generated by the DCGAN. Li et al. [29] have extended the idea presented in [23] by enlarging the convolutional kernels’ size and adding more convolution operations with different dilations within the DRI modules. The short-cut connection is made outside the DRI module, i.e., the residual connection is made between the input and the final output of the module, while in the previous version [23], different residual connections were made between the layers inside the DRI module. It is necessary to mention that the researchers in this field, both for the handcrafted features-based and deep learning-based methods, as described above, prefer to adopt, quasi unanimously, the supervised learning paradigm in order to tackle the HEp-2 images classification.

Supervised learning necessitates the presence of labeled images. Consequently, even though the discrimination performance that we can obtain by using this methodology remains remarkable, the necessity of constructing labeled datasets that contain a considerable number of images represents a serious concern. In fact, deep learning-based methods require the presence of thousands of images and the process of labeling by hands these images can eventually represent a quite onerous task in the future, when we will have to create more expanded datasets, which can be a drawback for this methodology. In our previous works [30,31], we have explored the possibilities afforded by the unsupervised learning approach for this topic. In the present work, we adopt the supervised learning methodology but in a quite different manner compared to what is actually done in the HEp-2 cell images classification literature. The principal contribution of this work is the use of the techniques afforded by active learning in order to drastically minimize the need of the labeled images (in proportion of the total number of images) while maintaining a comparable performance with the actual state-of-the-art methods.

For this purpose, we propose to use active learning coupled with transfer learning. Transfer learning consists of using an already trained network for a new task. Fine-tuning the trained network consists of updating its parameters using the new dataset. This technique was used for the HEp-2 cells classification by Phan et al. [32], who utilized a model, the VGG-16 network [33], that was previously trained on the ImageNet dataset. Some others methods utilize a pretrained network only as a feature extractor. The high-level features extracted from the pretrained CNN model are then used in order to train a multiclass SVM. In the HEp-2 cell classification literature, this technique (transfer learning without fine-tuning) has been adopted in different ways by the works discussed next. Lu et al. [34] have used a pretrained VGG-16 network as a feature extractor while Nguyen et al. [35] proposed the use of an ensemble of networks. Another work using this idea was presented by Cascio et al. [36] with the use of the AlexNet [37] as the feature extractor. For all of them, the discrimination was performed by a SVM, or by both a SVM and a k-nearest neighbor classifier, as done in [36].

An interesting transfer learning approach, named cross-modal transfer learning, was proposed by Lei et al. [38]. Cross-modal transfer learning consists of updating the parameters of the pretrained network (fine-tuning) in two steps: first, by using a quite small dataset, then, second, by performing the update on the targeted dataset, which is supposed to be much larger and more complex than the first one. With the particularity that the two datasets have to be similar, i.e., they have to share the same feature domain. In fact, most of the pretrained CNN models were trained on the ImageNet dataset, which contains images that are far different with the HEp-2 cell images. The idea of cross-modal transfer learning is like performing a prefine-tuning (on the small dataset) before a final fine-tuning (on the targeted dataset) in order to smooth the parameters’ updating process during the training. The authors in [38] have used ResNet-50 as the network to be fine-tuned. The small dataset utilized was the ICPR2012 dataset [2], and the targeted dataset was the ICPR2016 dataset [39], also known as the 13A dataset. Our method uses this cross-modal idea with a slight difference: we used the small dataset not in order to fine-tune an existing pretrained model, but in order to train our fully designed parallel deep networks.

Active learning regroups an ensemble of techniques whose aim is to minimize the data labeling burden while keeping the discrimination’s efficiency unchanged. The idea is to use the network in a set of iterations in order to select, among the totality of the data, only the samples that really need to be annotated and used it for training the final model. Different methods can help to select these data, methods based on uncertainty sampling [40] or query-by-committee [41]. Some other works have used active learning with deep learning [42,43,44,45] and some others have proposed different techniques, such as neural-like structures based on geometrical transformation model [46], in order to ensure the possibility of obtaining satisfactory results even with a small number of training examples. We aim to demonstrate in this work that active learning techniques can also be applied in the HEp-2 cell images classification and allow one to minimize the need of labeled data while maintaining a comparable discrimination performance with the state-of-the-art deep learning-based methods that utilize the totality of the labeled images.

The contributions of the present work can be summarized as follows. First, we propose a dynamic learning method that uses two deep residual networks with the same structure in parallel in order to specifically tackle the intraclass variations and interclass similarities present in most of the HEp-2 cell images datasets. Two-dimensional (2D) discrete wavelet transform (DWT) is performed over the input images. The first network takes the approximation coefficients as inputs and the second network takes the sum of all the details coefficients as inputs. The learning of the two networks is done in parallel and their high-level features are fused at the end of the networks in order to mix the different information extracted from the two inputs.

Second, we use the idea of cross-modal transfer learning in order to boost the efficiency of the active learning. The two parallel deep residual networks are first trained with a small HEp-2 dataset, the SNPHEp-2 [5], which contains only around 2000 images, in order to build our pretrained model. The third and principal contribution is to apply this pretrained model on our targeted large-scale HEp-2 dataset, which will be presented in details in Section 3, and utilize the uncertainty sampling from active learning in order to only select, in that dataset, the most informative data.

Finally, we systemically investigate the effectiveness of the proposed method and our experimental results demonstrate that: first, the proposed parallel residual networks are effective for the discrimination of the HEp-2 cells; second, by using an efficient transfer learning methodology (similar to cross-modal transfer learning), active learning can effectively help to minimize the burden of labeling images by hands during the dataset creation by allowing us to select only a few number of informative data that really need to be annotated while maintaining a quite fair performance on a very large-scale dataset. 

The remaining content of the paper is organized as follows. The next section (Section 2) presents in details each step of the proposed methodology. Section 3 presents the dataset, discusses the obtained results, and addresses a comparative study with the other supervised learning methods in the literature.

## 2. Proposed Method

**Figure 1:**
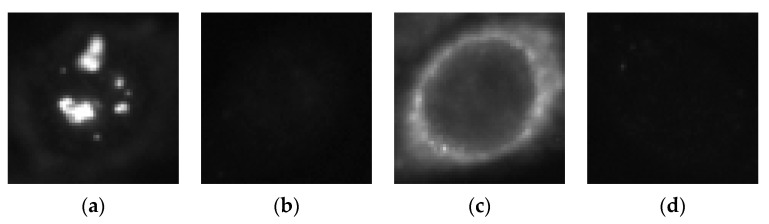
HEp-2 cellular images from the 13A dataset. (**a**) A positive intensity nucleolar cell; (**b**) a negative intensity nucleolar cell; (**c**) a positive intensity nuclear membrane cell and (**d**) a negative intensity nuclear membrane cell.

The first step of our method is to create the parallel networks that we will use for the transfer and active learning. HEp-2 cell datasets have the particularity of denoting a significant heterogeneity. This is caused by the existence of mainly two different levels of fluorescence illumination (also denoted as intensity levels). Images shown in Figure 1 illustrate the disparities caused by the inhomogeneous fluorescence illumination. These disparities are the intraclass variations and the interclass similarities. Intraclass variations denote the variations within the same cellular type. Figure 1a shows a randomly selected positive intensity nucleolar cell image, while Figure 1b shows a randomly selected negative intensity nucleolar image. We can remark the strong disparities in terms of intensity between the two images even though they belong to the same class (intraclass variations). The same dissimilarities can be noticed between the two images depicted in Figure 1c,d, in case of the nuclear membrane cell type.

Interclass similarities, on the other hand, denote the similarities that exist between the different classes. In fact, the images shown in Figure 1b,d exhibit strong similarities in terms of intensity even though they belong to two different cellular types. This heterogeneity-related problem really adds complexity in the HEp-2 cell classification task. As a matter of fact, different methods have been proposed in order to specifically classify the different fluorescence intensity [47,48]. Furtherly, Nigam et al. [49] have proposed to perform an intensity-based classification prior to the cell classification itself in order to alleviate the heterogeneity during the cell type classification. Our proposed parallel deep residual networks try to tackle this heterogeneity-related problem in one step (unlike in [49]) and by performing cell type classification (unlike in [47,48]).

### 2.1. Parallel Deep Residual Networks

**Figure 2:**
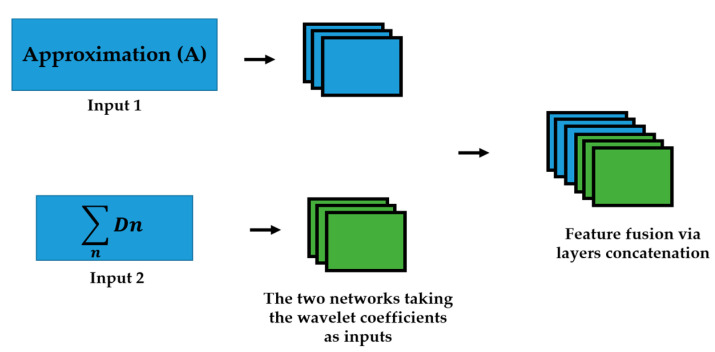
The approximation (A) and the sum of details, represented by D, are given to two networks. A feature fusion is performed in the late layers. Note that *n* here is 3 and represents the three details components.

We propose to use the different wavelet coefficients from the 2D-DWT decomposition as the inputs of different networks in parallel. This idea was fully discussed and its effectiveness demonstrated in [50]. We upgraded the idea by alleviating the learning (training) process by reducing the total number of needed networks and, consequently, the total number of the parameters to handle. The 2D-DWT in the first level produces 4 different matrices of coefficients. The approximation coefficients, which represent the low-frequency information of the inputs, and the three different details coefficients, which represent the high frequency components of the input signal. The three details coefficients are the horizontal, vertical and diagonal details. Unlike in [50], where four different networks were utilized in parallel for all the four coefficients, we sum up all the details coefficients in order to incorporate all the high frequency components in one single channel. Thus, as illustrated in Figure 2, two networks are trained in parallel: the first network takes the approximation coefficients as the inputs, and the second network takes the sum of all the three details coefficients as inputs.

**Figure 3:**
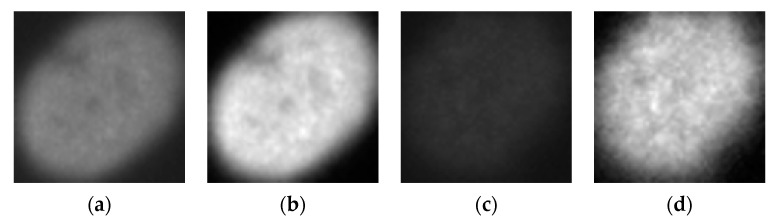
HEp-2 cellular images from the SNPHEp-2 dataset. (**a**) A positive intensity fine speckled cell; (**b**) its approximation coefficients; (**c**) a negative intensity fine speckled cell and (**d**) its approximation coefficients. Note the effective homogenization in terms of gray level intensity between the positive (**b**) and negative (**d**) images.

The approximation coefficients will bring a certain homogenization in terms of the intensity. This will drastically reduce the intraclass variations by forcing both the positive and negative intensity images to share a similar level of gray intensity. Images shown in Figure 3 illustrate the intensity-based homogenization produced by the approximation coefficients. Figure 3a shows a randomly selected positive intensity fine speckled cell image from the SNPHEp-2 dataset. Figure 3b shows its corresponding approximation coefficients (extracted from the first level of the 2D-DWT decomposition). Figure 3c shows a randomly selected negative intensity fine speckled cell image and Figure 3d shows its corresponding approximation coefficients. We can clearly remark the homogenization that occurred between the images in terms of the intensity of the gray level by comparing their approximation coefficients. This homogenization will drastically reduce the intraclass variations of the dataset.

**Figure 4:**
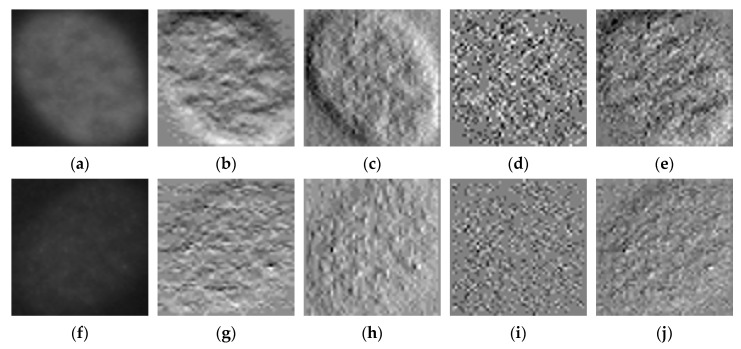
HEp-2 cellular images from the SNPHEp-2 dataset. (**a**) A positive intensity homogeneous cell and (**b**–**e**) its horizontal, vertical, diagonal details and their sum, respectively. (**f**) A negative intensity homogeneous cell and (**g**–**j**) its horizontal, vertical, diagonal details and their sum, respectively. The original images in (**a**,**f**) have a size of 112 × 112. Their respective detail coefficients in (**b**–**e**) and (**g**–**j**) are all downsized by half (56 × 56). All the images in the figure were identically resized for the purpose of visualization.

Secondly, the details coefficients will bring homogenization in terms of the geometrical shape of the cells. In fact, the details coefficients capture the high frequency components of the image, which means that all the gray variations inside the image can be highlighted. These small changes in intensity indicate the shape and the boundaries of the cells. Images depicted in Figure 4 illustrate how the high-frequency components can help to expose the cellular shape and boundaries from the positive and negative intensity images.

In Figure 4a, we show a positive intensity homogeneous cell. Images shown in Figure 4b–d represent its different details coefficients, the horizontal, vertical and diagonal details, respectively. The image shown in Figure 4e is the result of summing all the details. We can remark that the sum incorporates all the information from the three details coefficients. Similarly, in Figure 4f, we show a negative intensity homogeneous cell image. In Figure 4g–i, we show the three details coefficients and Figure 4j represents their sum. Note how the two sums (Figure 4e,j) highlight the homogeneous cell’s shape, boundaries and internal gray variations. Since these three elements differ from a cellular type to another, we can expect two main contributions from the sum of details. First, they will bring a certain heterogeneity between the classes by forcing all the negative intensity images to exhibit typical characteristics of their cellular type (shape, boundaries and gray variations). This will contribute to the reduction of the interclass similarities.

Consequently, as the second contribution, they will bring a certain intraclass homogenization by forcing the positive and negative intensity images from the same class to exhibit similar patterns (shape, boundaries and gray variations), as demonstrated by Figure 4e,j. This will contribute to the reduction of the intraclass dissimilarities, reduction also achieved by the approximation coefficients, as previously discussed. The approximation and the sum of details will be used to feed the two residual networks in parallel.

**Figure 5:**
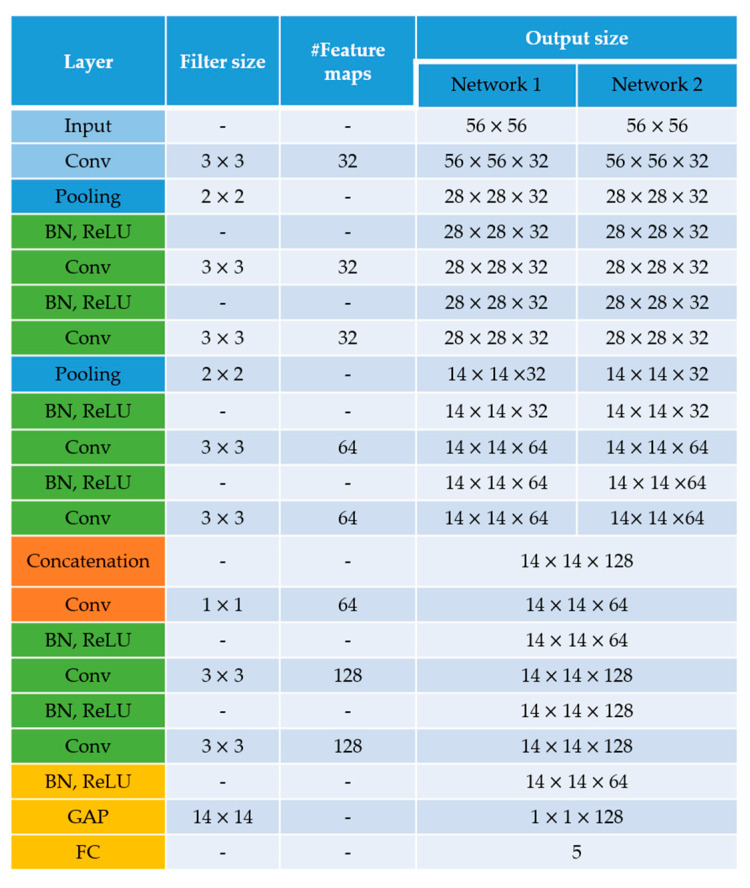
Architecture of the residual networks. The residual blocks are shown in green. Here, “Conv”, “BN”, “ReLU”, “GAP” and “FC” denote, respectively, the convolutional layer, the batch normalization layer, the rectified linear unit (ReLU) layer, the global average pooling layer and the fully connected layer. “Pooling” denotes the maximum pooling layer and “Concatenation” denotes the layer that performs feature concatenation from the two networks.

Figure 5 shows the architecture of the residual networks. Network 1 takes the approximation coefficients while Network 2 takes the sum of the details, as explained above. There are five residual blocks in total. Each network has two residual blocks and another is used after the feature fusion from the two networks. Every residual block has two convolutional layers, two rectified linear unit (ReLU) layers and two batch normalization layers [51].

Two main observations need to be made about the architecture in Figure 5. First, all the convolutional layers preserve the spatial dimensions of the input volume and only the pooling layers perform the spatial downsampling. Second, the feature fusion is performed by the 1 × 1 convolutional operation that directly follows the concatenation. After concatenating the layers from the two networks, we obtained a volume of size 14 × 14 × 128, which is then passed through the 1 × 1 convolutional layer whose purpose is precisely to mix (fusion) the information from the two networks. The output volume of the final residual block has the dimensions of 14 × 14 × 128. This volume is given to the global average pooling (GAP) layer in order to obtain the final one-dimensional feature vector of size 1 × 1 × 128. 

The feature vector will be given to a softmax classifier [52] that uses the function defined as follows:

(1) $$\sigma {\left(z\right)}_{j}=\frac{e^{z_{j}}}{\sum_{i=1}^{N}e^{z_{i}}},\;\;\mathrm{for}\;j=1,\ldots ,N,$$

where *N* is the number of the classes and the values *z* are the inputs of the softmax function. The values $\sigma {\left(z\right)}_{j}$ are the outputs of the function and represent the probabilities of every class. The parallel networks learn by back-propagating the error [53] and using the cross-entropy error function [52] defined by

(2) $$E=-\;\overset{N}{\underset{j=1}{\sum }}y_{j}\log\left[\sigma {\left(z\right)}_{j}\right],$$

where the values *y_j_* denote the actual labels of the *N* classes for a given data and the values $\sigma {\left(z\right)}_{j}$ are the ones computed using Equation (1).

These parallel networks will be first trained with a small dataset, which contains only around one thousand training instances. After this initial training process, the networks will be utilized as the pretrained model in order to perform transfer learning coupled with active learning on the targeted dataset, which contains more than sixty thousand instances. As previously discussed in Section 1, instead of using networks that were pretrained on ImageNet, as done by most of the works that utilize transfer learning [32,34,35,36], we propose to use our own networks, which are pretrained purposely by using the HEp-2 images.

**Figure 6:**
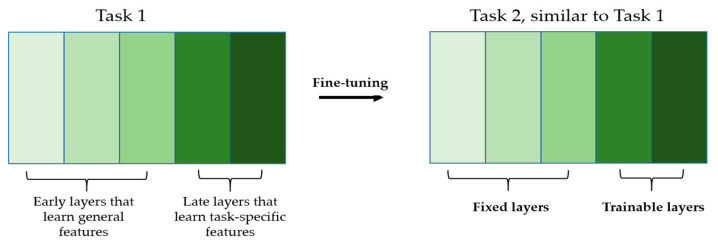
Illustration of fine-tuning. Only the late layers will be trainable during the fine-tuning. The different shades of green indicate how the specificity of the features increases layer after layer. From left to right, we have the general features to the task-specific features.

The advantage is that using a network that has previously seen similar images during transfer learning alleviates the parameters’ update. Since the two datasets share the similar image domains, we expect them to share many general characteristics. The early layers from the networks, which learn low-level and non-specific features, can be fixed during the fine-tuning and only the late layers, which learn domain-specific features, can be updated (see Figure 6 for the illustration). This will smooth and ease the first step of our active learning scheme consisting of fine-tuning the pretrained model on a very small number of selected data.

### 2.2. Active Learning Using the Pretrained Parallel Residual Networks 

Active learning aims to alleviate the labeling process by allowing one to select only the data that should be given to the learning model. The goal is to find which are the data that carry the most informative details that can help us to build the classifier. This selection can be done by finding the data for which the model is the most uncertain. This is referred as uncertainty sampling [40]. The idea is to find an uncertainty measure that can help to evaluate the confidence of the model and then use that measure in order to select only the data for which the model is the most uncertain. Select in order to annotate them. Instead of annotating (labeling by hands) all the available data, we can just annotate the ones for which our model is the most uncertain about. 

Different uncertainty measures have been utilized in the literature. Among the most used, we have the entropy [54], which can measure the certainty level of a classifier by using the probabilities (scores) that are attributed to each class. For a given instance x, the entropy can be evaluated by the following equation:

(3) $$entropy\left(x\right)=\;\overset{N}{\underset{j=1}{\sum }}p(y_{j}|x)\log p(y_{j}|x),$$

where the values $p(y_{j}|x)$ represent the classes’ probabilities (or classes’ scores) as outputted by the classifier for the data x and N is the number of classes. When the entropy is very high for a given data, it means that the classifier evaluates equally the different classes for that data. In other words, the classifier is uncertain about which class to assign to the data  x.

Another way to measure the uncertainty of a model is to compute its amount of confidence for a given instance. This method is referred as least confidence-based sampling [40,55]. For a given data x, the confidence C is given by

(4) $$C=\underset{j}{\mathrm{argmax}}p(y_{j}|x),$$

where the values $p(y_{j}|x)$ represent also the classes’ probabilities. We know that for every single instance, the classifier outputs a vector containing the probabilities associated to each class, represented here by $p(y_{j}|x)$. The confidence denoted in Equation (4) finds the maximum score, i.e., it finds the class for which the model assigns the maximum probability value. Which gives us the amount of confidence of the model for every single data. The idea is to select the data for which the model is the least confident, i.e., the data for which the values C are the lowest. In practice, we can sort the data according to their confidence C from the smaller to the larger, and then prioritize the annotation in that manner. Note that entropy also can be thought as the amount of confidence: when the entropy is high, the confidence is low, and vice versa. 

Another method, called the margin sampling [56], consists of computing the difference between the highest and the second highest scores in the vector of probabilities. In this case, the confidence C is given by

(5) $$C=\;p_{1}\left(y_{j}|x\right)-\;p_{2}\left(y_{j}|x\right),$$

where $p_{1}\left(y_{j}|x\right)$, and $p_{2}\left(y_{j}|x\right)$ are the highest and the second highest probabilities, respectively. Similar with the previous case, the data with the lower C values are prioritized for the labeling. The difference computed in Equation (5) enquires us about the confidence of the model. If its value is high, it means that one of the class has a much bigger probability compared to the other and, on the contrary, when its value is small, it means that the model evaluates equally the two classes, which means that the model is uncertain.

**Figure 7:**
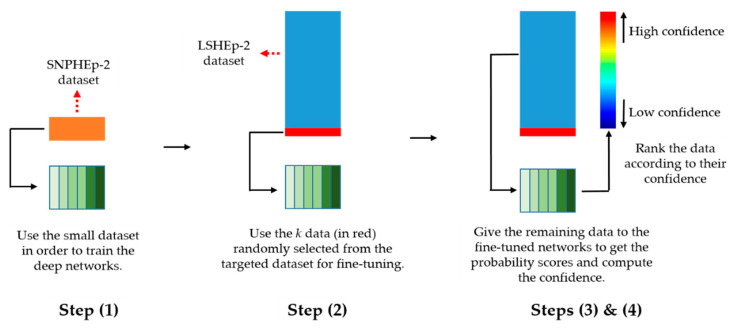

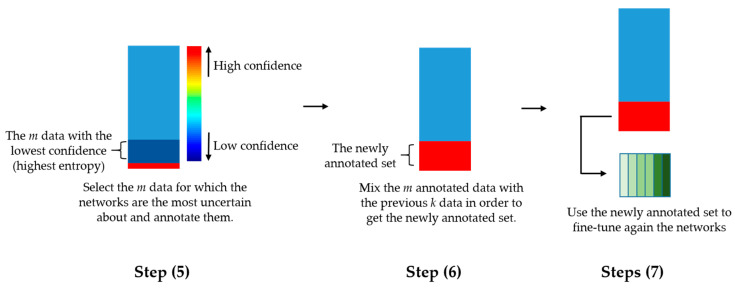
Illustration of the different steps of the proposed active learning scheme. LSHEp-2 stands for the large-scale HEp-2 dataset.

The least confidence and margin sampling methods work similarly and, unlike the entropy, are both less suitable for the multiclass classification. In fact, in Equation (5), only two classes are taken into account, while the entropy utilizes all the classes’ probabilities. In our case, we will adopt the entropy in order to evaluate the uncertainty of the deep parallel residual networks. Other uncertainty sampling methods can be found in [57,58,59]. For clarity, we summarize the different steps of the proposed active learning scheme in Table 1. Figure 7 illustrates these different steps. 

This process is repeated as much as possible and stopped until we reach our limitations in terms of labeling. Note that this iterative process can be continued until the totality of the data are labeled. However, in our work, we imposed to ourselves a limit in terms of the possibility of labeling. In fact, the goal of this work was to demonstrate that active learning-based labeling could really help to limit the need of labeled data while maintaining a fair performance. In our experiments, we explored different hypotheses concerning the limitations that we had in terms of labeling. For example, if we suppose that we can only label 10% of the 64,000 available data, we stop the process when we reach 6400 annotated data and evaluate the networks over the testing data. All the details concerning the parameters of the networks, the values *k* and *m*, and the datasets are discussed in the next section. 

## 3. Results

### 3.1. Datasets, Experimental Setup and Initial Learning Results

**Figure 8:**
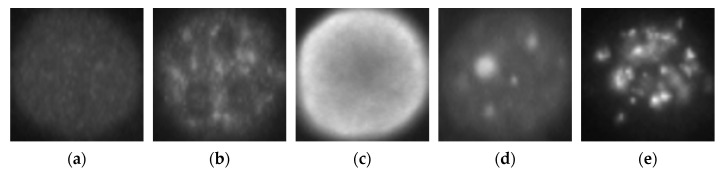
Example images from the SNPHEp-2 dataset. (**a**) The homogeneous, (**b**) the coarse speckled, (**c**) the fine speckled, (**d**) the nucleolar and (**e**) the centromere cells. The original size of the images is 112 × 112.

The deep residual networks were first trained using a relatively small dataset, as mentioned before. We adopted the SNPHEp-2 dataset here for this initial learning process. This dataset was presented by Wiliem et al. [5] and comprises five classes, which explains why the classification layer of the networks shown in Figure 5 had five neurons. The five classes (cell types) are the homogeneous, the coarse speckled, the fine speckled, the nucleolar and the centromere cells. The dataset contains 1884 data, divided into two sets: 905 images for training and 979 others for testing. Figure 8a–e shows one sample per class from this dataset. 

**Figure 9:**
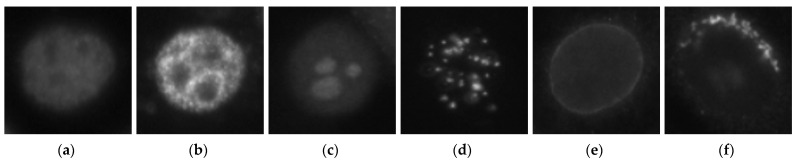
Example images from the large-scale HEp-2 dataset. (**a**) The homogeneous, (**b**) the speckled, (**c**) the nucleolar, (**d**) the centromere, (**e**) the nuclear membrane cells and (**f**) the Golgi cells.

Instead of using the ICPR2012 for the initial learning as done in [38], the choice of using the SNPHEp-2 dataset was justified by the similarities between this dataset and our targeted dataset. The targeted dataset is the large-scale HEp-2 (LSHEp-2) dataset, introduced by Qi et al. [60]. This dataset contains far more images (63,445) than the 13A dataset (13,596 images). The reason why we adopted the LSHEp-2 dataset is that we aimed to test the effectiveness of the proposed active learning scheme on a really big dataset for which labeling can really be burdensome. Furthermore, this dataset is more complex in terms of intraclass variations and heterogeneity compared to the others. The description of the LSHEp-2 dataset can be found in details in [60] and it can be downloaded at http://qixianbiao.github.io/HEp2Cell/ (accessed on 16 January 2021). Similar to the 13A dataset, it contains six classes: homogeneous, speckled, nucleolar, centromere, nuclear membrane and Golgi cells. Some examples of this dataset are shown in Figure 9.

By comparing the images in Figure 8 and Figure 9, we can remark that the two datasets share many similarities. We can expect that our networks will learn the general features shared by these two sets of images, which will allow us to only update the task-specific layers located at the end of the networks. In fact, two big changes can be remarked between the SNPHEp-2 and the LSHEp-2 datasets: firstly, the two speckled (fine and coarse) cells from the first were mixed to form only one cell type, the speckled cells, in the second. Secondly, the Golgi cells are absent from the SNPHEp-2. During the transfer learning process, we will remove the last layer containing five neurons and replace it by another one containing six. All the experiments were performed using TensorFlow on a computer with a Core i7 3.40 GHz processor, 8 GB of RAM and a NVIDIA GeForce GTX 1080 Ti GPU.

For the initial learning process, the hyperparameters were selected via cross-validation using the five different validation folds of the SNPHEp-2 dataset. The original images had different sizes (average around 90 × 90) and were all upsized using bicubic interpolation to 112 × 112 in order to fit into our designed architecture. Note that after the DWT decomposition, the coefficients at the first level have all the size of 56 × 56. In order to maximize the learning capacity of the networks, data augmentation was applied over the SNPHEp-2 dataset. It consists of cell rotation, with a step of 18° in a quadrant of 360°, as proposed in [16,25]. This rotation increases the original training set by a factor of 20. 

**Figure 10:**
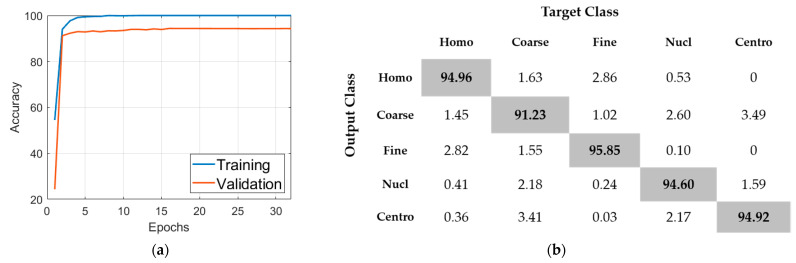
Results of the initial learning process: (**a**) the progression of the accuracy of training and validation; (**b**) the confusion matrix (total accuracy of 94.31%). “Homo”, “Coarse”, “Fine”, Nucl” and “Centro” stand for homogeneous, coarse speckled, fine speckled, nucleolar and centromere, respectively.

The learning rate is set to be 0.001 and training is terminated when the validation loss did not surpass the reached minimum 5 times in a row. For the initial learning (with data augmentation), 32 epochs were necessary to terminate the training process (see Figure 10a). The classification results of this initial learning are shown in the confusion matrix depicted in Figure 10b. The accuracy over the validation set, as we can see in Figure 10, was about 94%. We recall here that the purpose of this initial learning is just to generate a pretrained model that will be used later for the transfer learning. For further details about the effectiveness of the dynamic learning afforded by the wavelet coefficients, readers are invited to check our previous work [50] where we have presented a detailed discussion about it. 

After we had our pretrained model in hands, we could utilize it for the active learning over the LSHEp-2 dataset. For all the fine-tuning procedures, we had fixed all the layers before the second residual block, which means from the first convolutional layer to the second pooling layer (see Figure 5 for the architecture). The second residual block is set to be trainable because we want the networks to extract features that are specific to our main dataset before the feature fusion (layer concatenation). The final layer was changed to have six neurons, according the six classes of the LSHEp-2 dataset. The same learning approach was used for all the fine-tuning processes: a learning rate of 0.001 was utilized, training is stopped when the loss does not decrease five times in a row.

As said before, the LSHEp-2 dataset contains 63,445 images. A 80–20% splitting was performed, which gives 50,758 images for training, and 12,687 for testing. The labeling limitations that we imposed to ourselves only concern the training set (the testing set was just used for validation, not for fine-tuning). We principally tested our method with the limitation of being able to annotate only 20% of the training set, which gives a total of 10,152 images. The value *k* was set to be 1500, i.e., 1500 images (around 15% of the 10,152) were first selected randomly in order to perform the first fine-tuning. Additionally, then, the value *m* was set to be 1000, which means that we selected the first 1000 data in the ranking performed in step 4 (see Table 1) in every labeling iteration. We stop the labeling process after we annotated the totality of the 10,152 images. Note that the same kind of process was repeated for any level of limitation (the number *k* being 15% of the limitation and *m* being 10%).

The results are shown in two categories: the results without cross-modal transfer learning and the ones with cross-modal transfer learning. The first category designates the case where we did not use the initial learning for building the pretrained model. We just trained the networks by using directly the first *k* images from the main dataset. The second category designates the proposed scheme, where initial learning with a small dataset was used before fine-tuning with the main dataset. In every category, we show two cases for the results: using random sampling and using active learning-based sampling. In other words, and with the case of 20% of the limitation, random sampling designates the fact of selecting randomly 20% of the training images in order to train the networks while active learning-based sampling designates the fact of using active learning techniques for the selection of 20% of the training images.

Note that for all the cases where active learning was involved, we did not show the loss and accuracy progression since several different learning procedures were conducted in every labeling iteration (many fine-tunings). In this case, showing the loss and accuracy evolution was meaningless. On the other hand, these evolutions are shown for the cases that did not involve active learning, where only one single training procedure was performed. For simplicity, the different cases were designated by their short names shown in Table 2.

For other limitations (5%, 10%, 30%, 40% or even 100% of the training set), the results were summarized and discussed later. The case of 100% means that we could utilize the totality of the training data without any limitation. Note that the datasets (SNPHEp-2, 13A and LSHEp-2) all exist in a labeled form. The labeling limitations suggested in this work are indicative of the potential afforded by active learning and were used here in order to demonstrate its effectiveness.

### 3.2. Results without Cross-Modal Transfer Learning

**Figure 11:**
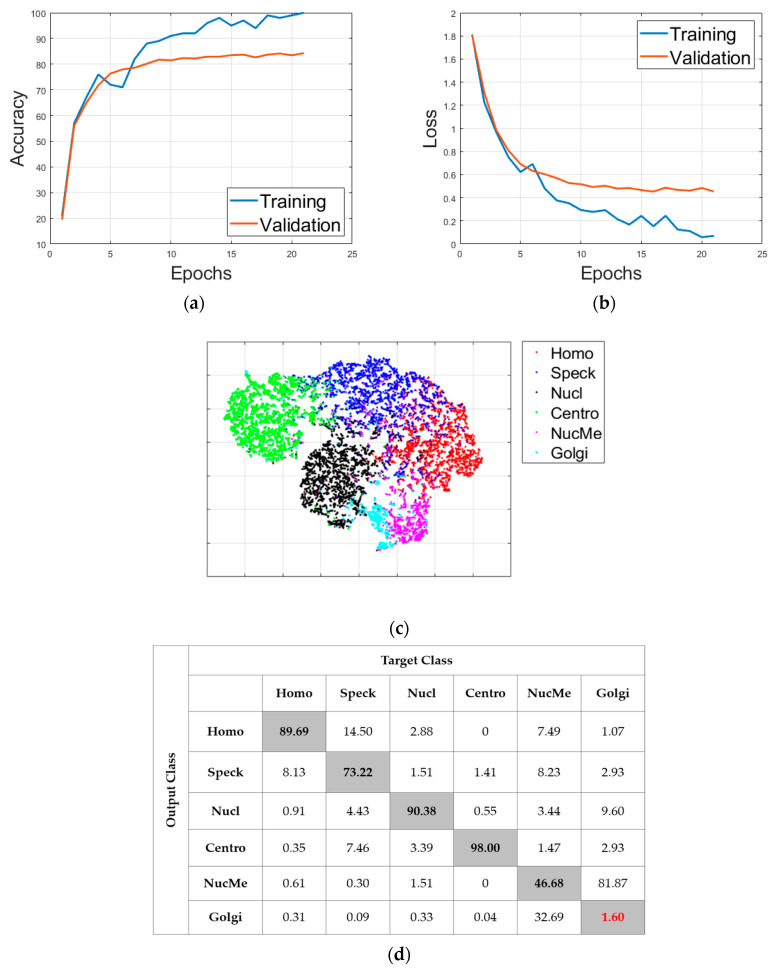
Classification results of the “RS” case: (**a**) accuracy, (**b**) loss, (**c**) features’ visualization and (**d**) confusion matrix.

As said before, all of the following results concern the case of 20% of the limitation. Figure 11 shows the detailed results of the “RS” case. This case just consists of selecting randomly 20% of the images and uses them to train the deep networks. Figure 11a shows the accuracy evolution over the training and validation sets (21 epochs). Figure 11b shows the loss evolution for the two sets. Figure 11c shows the visualization of the high-level features learned by the deep networks. All the visualizations here are obtained using the t-distributed stochastic neighbor embedding (t-SNE). Finally, Figure 11d shows the confusion matrix of the classification over the validation set. In all the confusion matrices shown here, “Homo”, “Speck”, “Nucl”, “Centro”, “NucMe” and “Golgi” refer to the homogeneous, speckled, nucleolar, centromere, nuclear membrane and Golgi cells, respectively.

By analyzing the results, we could see that selecting randomly the data did not help for the generalization over the validation set. Two main observations can be highlighted from these results. First, there was a clear difference between the mean class accuracy (MCA) and the average classification accuracy (ACA). The MCA was 66.59% and the ACA was 81.13%. The ACA, which computes the overall accuracy by dividing the number of correctly classified data by the total number of data, appeared to take advantage of some of the classes that were very well discriminated. In particular, the nucleolar (90.38%) and the centromere (98.00%) contributed highly to establish the ACA in a very pleasant level. 

On the other hand, the MCA, which computes the mean of all the classes’ accuracies, was hugely impacted by the poor classification accuracy of the Golgi and nuclear membrane cells. As part of the second main observation, as we can also remark in Figure 11c by analyzing the visualization of the features, there was an extreme confusion between the two cells’ clusters (Golgi in magenta and nuclear membrane in cyan). In fact, only 1.6% of the Golgi were well classified, while 81.87% of them were misclassified as the nuclear membrane, as we can see in the confusion matrix depicted in Figure 11d. Also, only 46.68% of the nuclear membrane were well classified. The two cellular types were certainly the most complicated to discriminate. The first reason is that both types are always under-represented among the available data in all the existing datasets. There were only 375 Golgi and 814 nuclear membrane instances in the training set, while all the others cell types contained each at least 2100 images. The second reason is the complexity of their shape. Having the possibility of using only 20% of the training set, which diminishes again their number among the selected data for training, contributes to making the discrimination harder for the two cells. This pointed fact represents the principal observation of the present work. While having a limited number of labeled data in hands, the classification of these two cell types (Golgi and nuclear membrane) becomes really complex.

**Figure 12:**
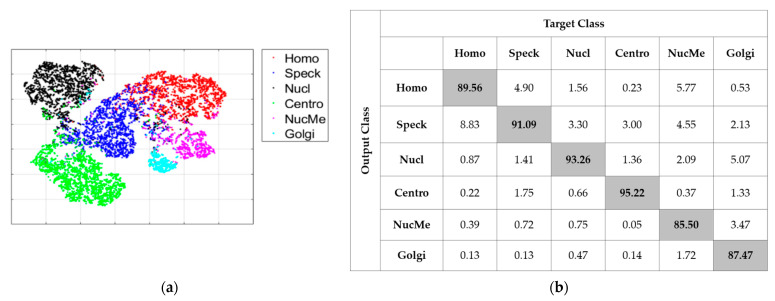
Classification results of the “AL” case: (**a**) features’ visualization and (**b**) confusion matrix.

Figure 12 shows the results for the second case (“AL”). As for the first one, this case consists of not using the initial learning but, on the contrary, selects 20% of the data with active learning. The MCA for this case was 90.35% and the ACA was 91.51%. As we can notice in the confusion matrix in Figure 12b, most of the cells maintained a quite fair classification result. Furthermore, and even more importantly, the huge confusion between the Golgi (87.47% of accuracy) and nuclear membrane (85.50%) had clearly diminished. The visualization in Figure 12a shows a noticeable separation of the two clusters compared with Figure 11c. We could notice, in these results, the improvement afforded by the active learning-based selection. By selecting, precisely for annotation, the data for which the networks were the most confused about, active learning decreased the discrimination’s complexity of the most difficult cells. At the same time, it maintained a good accuracy for the other cellular types.

### 3.3. Results with Cross-Modal Transfer Learning

**Figure 13:**
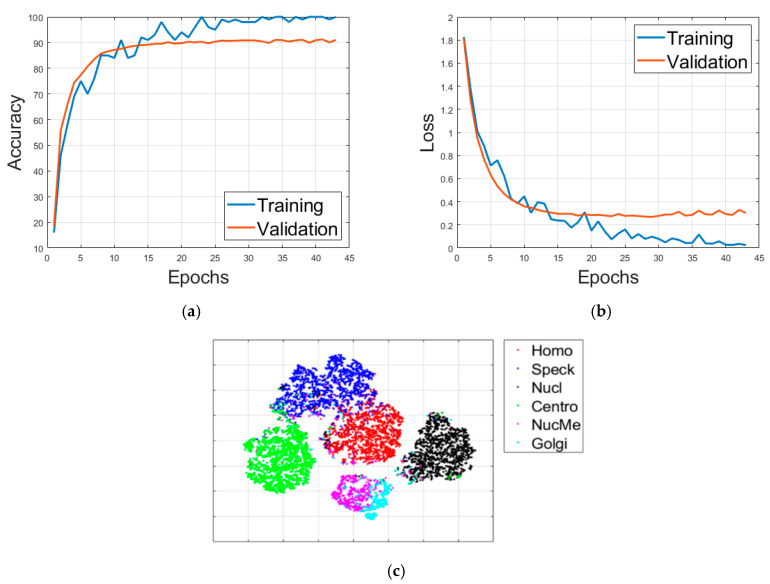

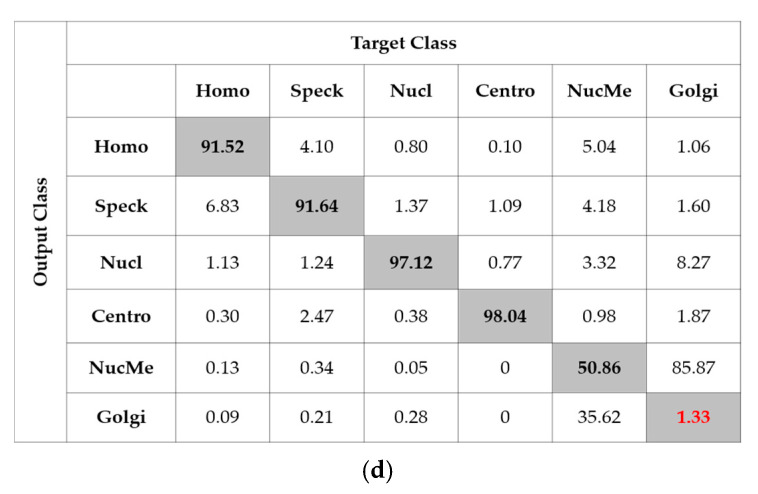
Classification results of the “IN_RS” case: (**a**) accuracy, (**b**) loss, (**c**) features’ visualization and (**d**) confusion matrix.

Here, we discuss the results obtained when an initial learning was performed in order to build the pretrained model. The first case (“IN-RS”) consists of selecting the 20% randomly in order to perform fine-tuning. In Figure 13a–d, we show, respectively, the accuracy, loss, visualization of the features and confusion matrix.

Here also, we could notice how the extreme confusion remained present even after we applied cross-modal transfer learning. The MCA was 71.75%, which was better than the “RS” case. However, the poor accuracy (1.33%) of the Golgi really pulled down the MCA, even though the other cells accomplish excellent accuracies (50.86% for the nuclear membrane). The ACA was 87.54% for this case. Note that the initial learning process increased the overall accuracy (the ACA goes from 81.13% to 87.54% between “RS” and “IN-RS”), but not for the two most difficult cellular types. 

**Figure 14:**
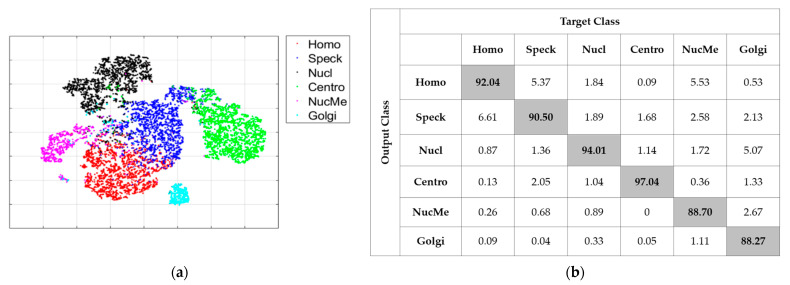
Classification results of the “IN-AL” case: (**a**) features’ visualization and (**b**) confusion matrix.

The second case, denoted as “IN-AL”, consists of selecting the 20% with active learning in order to perform fine-tuning. In Figure 14a,b, we have, respectively, the visualization of the features and the confusion matrix. As for the “AL” case discussed previously, we can notice how active learning permitted to tackle the extreme confusion between the Golgi and the nuclear membrane cells. This can be noticed in Figure 14a with the two clusters being completely disjoint, and in Figure 14b, where we see that the two cells accomplish reasonable accuracy. For this case, the MCA was 91.76% and the ACA was 92.77%. The most important observation here was that active learning strongly minimized the divergence between the two metrics by assuring a quite fair discrimination for all of the cellular types. Note that all of these results were obtained by using only 20% of the available training instances in the large HEp-2 dataset.

**Figure 15:**
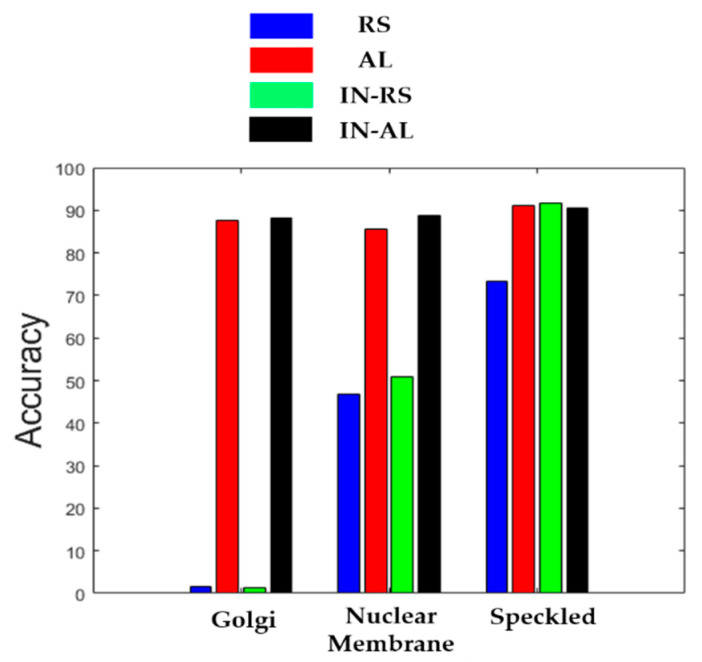
Classification accuracy of the three most difficult cells (the Golgi, the nuclear membrane and the speckled).

In Figure 15, we show the classification accuracies of the three most difficult cells for the four cases (“RS”, “AL”, “IN-RS” and “IN-AL”). We can notice how both cases that use active learning, by allowing one to prioritize the annotation of the most difficult cells, “correct” the classification accuracies of the cases without active learning (especially for the Golgi and nuclear membrane).

**Figure 16:**
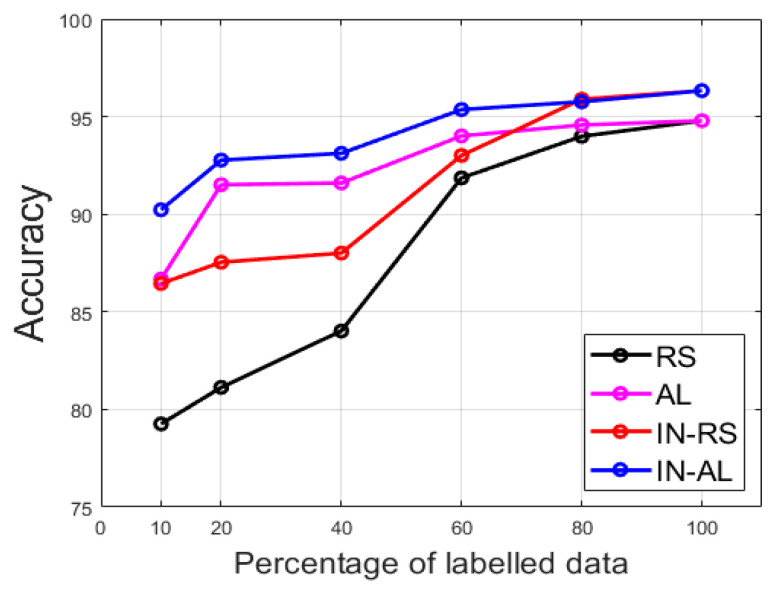
Evolution of the accuracy with other limitations in terms of labeling. The accuracy is shown for the 4 different cases discussed above.

In Figure 16, we show the summary of the results (ACA) for the others limitations. We show for the 10%, 20% (discussed in details previously), 40%, 60%, 80% and 100%. As explained before, the 100% case refers to the fact of using all the available training data. There was no active learning process in this case since all the data were supposed to be labeled. In this case, and only for this case, random sampling and active learning results were the same, as we can notice in Figure 16. For all the other limitations, we can see how active learning can help to achieve satisfying results even though we do not have access to the totality of the training data. In fact, for all the limitations, active learning-based labeling provided accuracies that were superior to 90%. It is only for the case of 10% limitation that active learning without cross-modal transfer learning (“AL”) achieved 86.68% (see Figure 16). However, this result could be significantly improved by using cross-modal transfer learning, as proposed in this work. In that case (“IN-AL”), the accuracy for the 10% limitation reached 90.23%. In other words, active learning coupled with cross-modal transfer learning allows one to achieve satisfying discrimination results even with a few number of labeled data in hands.

**Figure 17:**
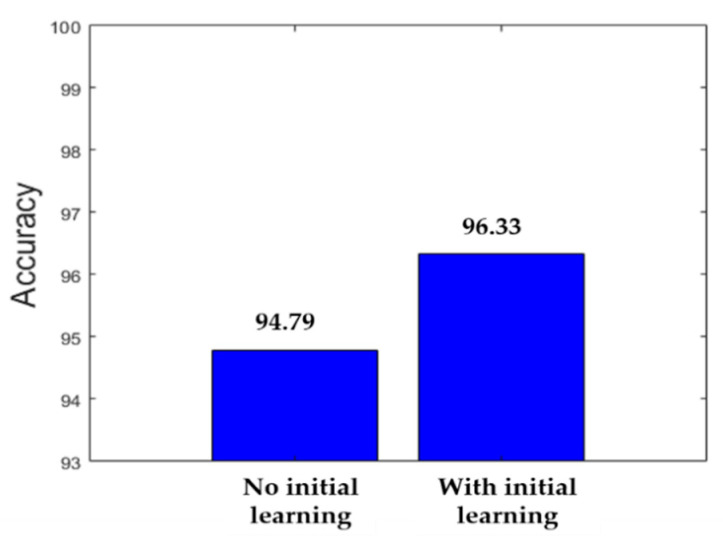
Accuracy improvement with the initial learning in the case of 100% of the training data available.

In order to show the contribution of cross-modal transfer learning, we show in parallel the classification accuracy when there was no limitation (100% of training data available) for the case where no initial learning was performed and for the case where we used the small dataset in order to build the pretrained model and perform cross-modal transfer learning. Figure 17 shows the comparison. We can remark how using the initial learning really improved the overall accuracy of the networks. 

In Table 3, we show the results of some different approaches. Note that most of the approaches were proposed for either the ICPR2012 or 13A datasets. In this comparative study, we tried to see how these approaches reacted on the bigger and more complex LSHEp-2 dataset. Note also that none of these approaches utilized active learning. The comparisons were done with the use of the totality of the training data and the aim of the comparative study was more to demonstrate the effectiveness of our deep parallel networks and the contribution of the cross-modal transfer learning process. 

Similar hyperparameters’ settings were used for the training procedures of the models used here for comparison. The learning rate was set to 0.001 and training was terminated after the loss plateaus for 10 consecutive epochs. We used a mini-batch of 128. Except in the case of our method, for all the methods involving transfer learning, the parameters were updated for all the layers, since all of the pretrained models were previously trained on ImageNet. Data augmentation, using the same technique as previously explained, was applied for the two methods that did not involve transfer learning, since the models were trained from the scratch.

As we can see in Table 3, most of the approaches using the models that were pretrained on ImageNet performed less than the ones that used cross-modal transfer learning. The state-of-the-art method in [38] utilize ResNet-50 but with an initial learning performed by using the ICPR2012 dataset, while their targeted dataset is the 13A. Our proposed method uses the deep parallel networks and the SNPHEp-2 dataset was utilized for the initial learning. Another state-of-the-art method is the DRC-Net [25], which achieves 94.15% on the LSHEP-2 dataset. We can notice that the accuracies shown in Figure 16 are similar with these state-of-the-art performances. Active learning coupled with cross-modal transfer learning allows one to achieve pleasant performance even with limitations in terms of labeling.

Note that models like VGG-16, VGG-19 and AlexNet require a substantial memory because of the enormous number of parameters generated by the fully connected layers at the end of the network. The residual networks (our parallel networks and the ResNet-50) also require a lot of memory but the computational complexity is far less compared to the other networks. This is explained by the global averaging pooling layer, which efficiently minimizes the computational complexity by diminishing the total number of parameters of the networks. Another important point to note is that all the methods used in the comparative study in Table 3 necessitate only one single training procedure. On the other hand, every case that involves active learning in our method necessitates several training procedures because of the iterative labeling process. This fact can be considered as the principal limitation of our method, as the cascade of training procedures elongate the time needed to build the final model. However, as previously discussed, our aim was to demonstrate that we could achieve quite pleasant performance with only a limited number of labeled data in hands. 

## 4. Conclusions

The automatic classification of the HEp-2 cell images represents an essential step in the production of the computer-aided diagnosis systems. The quasi-totality of the approaches in the HEp-2 cell classification literature prefer to address this problem by adopting the supervised learning approaches. Deep learning-based supervised learning necessitates the availability of thousands of images labeled by hands by the biological experts. This labeling process, especially in the case of big datasets, can represent a time consuming and burdensome task. Our work aimed to present a supervised learning approach that can minimize the need of the labeled data, thus, minimize the cost related to the labeling process.

In this purpose, we proposed a methodology that utilizes active learning coupled with transfer learning. We first proposed deep parallel networks that tackle the inter-class variations of the HEp-2 datasets. We then proposed an initial learning process using these networks over a quite small and labeled dataset in order to have a pretrained model. This pretrained model was then used with active learning on the targeted dataset, which was much bigger and more complex than most of the popular HEp-2 datasets, in order to select only the data that really need to be labeled. 

The proposed methodology alleviated the labeling process by allowing the networks to achieve quite satisfying discrimination results even with a limited number of labeled data. In fact, by using only 20% of the available training examples, our parallel networks achieved a mean class accuracy (MCA) of 91.76% while the average classification accuracy (ACA) reached 92.77%. These results were similar with the performance reached by the actual state-of-the-art methods, which, on the other hand, utilize the totally of the available training data. We demonstrated that this was made possible by the fact that active learning allows to prioritize the labeling of the most difficult cells and, thus, allowed the networks to maintain a good discrimination performance for each one of the cellular types. We demonstrated that the proposed methodology allowed us to minimize the divergence between the MCA and ACA by assuring a better discrimination for the Golgi and nuclear membrane cells. Since the need of data increased exponentially with the deep learning-based applications, we believed that the present work could be useful in the future in order to somehow ease the manual labeling task. The next step of our work consists of exploring different selection scenarios that do not necessitate training the model several times in order to reduce the computational complexity.

## Figures and Tables

**Table 1:** The different steps of our active learning scheme.

Step No.	Actions	Comments
1	We train our networks using the small dataset.	The small dataset used here is the SNPHEp-2 dataset, which contains around 1000 images for training.
2	Using the targeted dataset, we select randomly and label *k* samples. We fine-tune the networks by using these *k* samples as the training data. As described in Section 2.1, the early layers remain fixed and we only update the late layers.	Note that by choosing the number *k*, we select randomly (as opposed to select by using active learning) the data to label. In fact, we want this number *k* to be as small as possible, in order to not complicate the labeling process. This is made possible by the pretraining made in step 1 using the small dataset.
3	We use the fine-tuned networks over all the remaining data in order to get their probability scores. We compute the confidence (entropy) using Equation (3) for each data.	Equations (4) and (5) can also be used to estimate the confidence.
4	We rank the data according to their confidence, from the lowest to the highest.	Note that in Figure 7, we show the data with the lowest confidence (highest entropy) in the bottom for the illustration purpose.
5	We select the first *m* data in the ranking in step 4 and annotate them. These are the data for which the networks are the most uncertain about.	The number *m* is chosen according to the limitations that we have in terms of manual labeling.
6	The newly annotated data in step 5 are mixed with the *k* data that were previously labeled in step 2 in order to create the newly annotated set.	The newly annotated dataset contains now k + m data.
7	We fine-tune again the networks using this newly annotated dataset	After this step, we get back to step 3 (use the newly fine-tuned networks to compute the scores and the confidence).

**Table 2:** The different cases used during the experiments.

Case Name	Comments
*RS* (random sampling)	No initial learning, and selection using *random sampling*.
*AL* (active learning)	No initial learning, and selection using *active learning*.
*IN-RS* (random sampling with cross-modal transfer learning)	Initial learning involved, and selection using *random sampling*.
*IN-AL* (active learning with cross-modal transfer learning)	Initial learning involved, and selection using *active learning*.

**Table 3:** Comparative study using the LSHEp-2 dataset.

Methods	Accuracy (ACA)
Handcrafted features-based approach [60]	86.61%
LeNet-5-like CNN without transfer learning [16]	88.75%
VGG-16-like network without transfer learning [21]	90.23%
Transfer learning using the pretrained VGG-19	91.57%
Transfer learning using the pretrained AlexNet	92.41%
Transfer learning using the pretrained VGG-16 [32]	92.89%
DCR-Net [25]	94.15%
Transfer learning using the pretrained ResNet-50	94.36%
Our proposed deep parallel residual nets without cross-modal transfer learning	94.79%
Cross-modal transfer learning using ResNet-50 [38]	95.94%
Our proposed deep parallel residual nets with cross-modal transfer learning	96.33%

## Data Availability

All the data used for the experiments are publicly available can at http://qixianbiao.github.io/HEp2Cell/ (accessed on 16 January 2021).
